# Identification of a Novel Disulfidptosis-Related Five-Gene Signature for Prognostic Prediction and Immune Characterization in Esophageal Cancer

**DOI:** 10.3390/biology15070545

**Published:** 2026-03-28

**Authors:** Yiru Chen, Xuefeng Li, Hui Jiang, Xiaohui Liu, Nan Ma, Xuemei Wang

**Affiliations:** 1 State Key Laboratory of Digital Medical Engineering, School of Biological Science and Medical Engineering, Southeast University, Nanjing 210096, China; 220234830@seu.edu.cn (Y.C.);; 2 Joint Graduate School of Southeast University-Monash University, Suzhou 215123, China

**Keywords:** esophageal cancer, disulfidptosis, prognostic signature, tumor microenvironment, nomogram

## Abstract

Esophageal cancer is a highly aggressive malignancy with a poor prognosis. More precise prognostic biomarkers are therefore needed. Disulfidptosis is a recently identified form of regulated cell death driven by disulfide stress. It has been implicated in tumor progression. However, its prognostic role in esophageal cancer remains largely unexplored. This study aimed to develop a disulfidptosis-related gene signature for risk stratification and outcome prediction in esophageal cancer patients. Based on 23 disulfidptosis-related genes, consensus clustering was performed to identify molecular subtypes. Differentially expressed genes (DEGs) between subtypes were subjected to functional enrichment, immune microenvironment, and drug sensitivity analyses. Univariate and multivariate Cox regression were used to construct a prognostic risk model, which was evaluated using time-dependent receiver operating characteristic (ROC) curve and Kaplan–Meier analysis. A clinical nomogram integrating the risk score and clinicopathological factors was developed and validated. Two distinct disulfidptosis-related subtypes were identified, showing significant differences in gene expression, immune infiltration, and stromal scores. A total of 1080 DEGs were enriched in pathways related to epidermal differentiation, NRF2 signaling, and glucocorticoid receptor activity. A five-gene prognostic signature was established and effectively stratified patients into high- and low-risk groups. The risk model exhibited strong discrimination for 1-, 3-, and 5-year overall survival outcomes. The predictive accuracy was further maximized through an integrated clinical nomogram, which achieved an outstanding area under the curve (AUC) of 0.94 for 5-year survival predictions. Drug sensitivity analysis revealed subtype-specific therapeutic vulnerabilities, supporting potential precision treatment strategies. This study proposes a novel disulfidptosis-related five-gene signature and nomogram that robustly predict prognosis in esophageal cancer. The findings highlight the clinical relevance of disulfidptosis in tumor biology and offer a potential tool for risk stratification and personalized therapeutic decision-making.

## Simple Summary

Esophageal cancer is a highly aggressive disease with a low survival rate. This study aimed to improve these predictions by focusing on specific genetic characteristics and disulfidptosis. We analyzed the genetic makeup of tumors and identified two distinct patient groups. We also found five specific genes strongly linked to patient survival. We used these five genes to develop a risk-scoring tool. This tool accurately forecasts long-term patient outcomes. Furthermore, we found that different risk groups respond better to specific medications. This tool gives us a clearer understanding of tumor biology. It allows for more accurate predictions of disease progression. This research can provide new strategies to benefit patients by supporting personalized medicine. Healthcare providers can also use it to choose the most effective treatments for precisely personal medicine, which can ultimately improve the treatment efficacy and the quality of life for patients with esophageal cancer.

## 1. Introduction

Esophageal cancer remains one of the most formidable challenges in modern oncology, representing a significant cause of morbidity and mortality worldwide. According to the most recent global cancer statistics from GLOBOCAN 2022 and updated reports in 2024, esophageal cancer ranks as the seventh most frequently diagnosed malignancy and the sixth leading cause of cancer-related death globally [1]. The disease demonstrates a striking geographical distribution and a high mortality rate. This poor prognosis is largely attributed to its asymptomatic progression in the early stages, coupled with the rapid biological aggressiveness of advanced tumors.

Histologically, esophageal cancer is primarily categorized into esophageal squamous cell carcinoma and esophageal adenocarcinoma. These subtypes exhibit fundamental differences in etiology, pathogenesis, and epidemiology. Esophageal squamous cell carcinoma remains the dominant global subtype, accounting for nearly 85–90% of cases [2] and is strongly linked to lifestyle factors such as tobacco and alcohol use, nutritional deficiencies, and the consumption of hot beverages [3].

Despite significant advances in medical technologies in recent years, the overall prognosis for esophageal cancer remains unfavorable. The global 5-year survival rate hovers around 20%, which is markedly lower than that of other common malignancies such as colorectal or breast cancer [4].

For decades, the clinical management of esophageal cancer has relied heavily on the TNM staging system established by the American Joint Committee on Cancer. While this system provides a standardized anatomical framework for classifying tumor extent, anatomical staging alone is insufficient for predicting individual patient outcomes. Notably, patients with identical TNM stages often exhibit vastly divergent clinical trajectories. The TNM staging system relies primarily on anatomical features and fails to reflect the intrinsic molecular heterogeneity of tumors. In clinical practice, patients at the same stage often exhibit vastly different responses to radiotherapy, chemotherapy or immunotherapy. While some patients achieve a pathological complete response, others experience rapid recurrence or metastasis [5]. In the era of precision medicine, mining novel molecular markers based on bioinformatics analysis and constructing multi-gene prognostic models have become key approaches to breaking through the bottleneck in the diagnosis and treatment of esophageal cancer.

Over the past several decades, cancer therapy has primarily focused on the induction of apoptosis. However, tumor cells often develop resistance to pro-apoptotic treatments due to the overexpression of anti-apoptotic proteins and the inactivation of apoptotic executioners [6]. To circumvent this obstacle, researchers have shifted their focus toward non-apoptotic forms of regulated cell death, such as necroptosis, pyroptosis, ferroptosis, and cuproptosis. These novel forms of cell death are not only mechanistically distinct from apoptosis but also often closely associated with metabolic reprogramming in tumor cells. In 2023, Liu et al. [7] defined a novel type of cell death induced by disulfide stress, termed disulfidptosis. This discovery has fundamentally reshaped our understanding of the survival mechanisms of tumor cells under glucose starvation conditions. The core mechanism of disulfidptosis can be summarized as follows: in cancer cells with high *SLC7A11* expression, glucose starvation leads to depletion of NADPH, which in turn causes abnormal accumulation of intracellular disulfides. This ultimately results in aberrant disulfide cross-linking of actin cytoskeleton proteins, leading to cytoskeleton collapse and cell death [8]. Existing studies indicate that dysregulation of disulfidptosis-related genes may promote the progression of various cancers, such as lung cancer [9] and liver cancer [10]. For instance, increased expression of the disulfidptosis-associated gene *SLC7A11* has been linked to chemoresistance and poor prognosis in cancer [11]. However, a comprehensive analysis of the prognostic significance of disulfidptosis-related gene signatures in esophageal cancer is currently lacking.

Therefore, this study aimed to identify a disulfidptosis-related gene signature to predict clinical outcomes in esophageal cancer. Using The Cancer Genome Atlas (TCGA) database [12], we constructed a prognostic risk model and further explored the tumor microenvironment and drug sensitivity profiles associated with different disulfidptosis-related subtypes. Elucidating the role of disulfidptosis-related genes in esophageal cancer may not only improve risk stratification and prognostic prediction but also reveal novel therapeutic targets and strategies for this devastating malignancy.

Constructing a precise prognostic model for esophageal cancer requires a thorough analysis of genes involved in the core process of disulfidptosis. This study focuses on 23 key genes: *SLC7A11*, *CD2AP*, *PDLIM1*, *ACTN4*, *MYH10*, *IQGAP1*, *FLNA*, *TLN1*, *MYL6*, *ACTB*, *CAPZB*, *GYS1*, *NDUFA11*, *NCKAP1*, *NDUFS1*, *RPN1*, *NUBPL*, *SLC3A2*, *INF2*, *MYH9*, *FLNB*, *DSTN*, and *LRPPRC*. These genes constitute the complete execution network of disulfidptosis (Table 1).

## 2. Materials and Methods

### 2.1. Data Acquisition and Processing

RNA-sequencing expression profiles and corresponding clinical follow-up data for esophageal cancer were retrieved from the publicly available TCGA database. To ensure data comparability, the RNA-sequencing data were downloaded in the Fragments Per Kilobase of transcript per Million mapped reads (FPKM) format. The FPKM metric intrinsically normalizes the raw read counts by accounting for both sequencing depth and gene length. To minimize statistical noise and reduce the burden of multiple hypothesis testing, genes with zero or persistently low expression levels across the majority of samples were rigorously filtered out. Subsequently, the FPKM values were log_2_-transformed (log_2_(FPKM + 1)) to stabilize the variance and make the data tend towards a normal distribution. In this transformation, a pseudocount of 1 was added to avoid logarithmic transformation of zero values and to effectively dampen the inflated variance of extremely low-expressed genes, thereby ensuring the dataset more stable and normally distributed. Samples lacking complete overall survival time or survival status information were strictly excluded to ensure analytical accuracy and prevent confounding biases.

### 2.2. Consensus Clustering Based on Disulfidptosis-Related Genes

To identify potential molecular subtypes based on distinct disulfidptosis features, unsupervised consensus clustering was performed on the esophageal cancer patient cohort using the “ConsensusClusterPlus” R package (version 1.74.0) [35]. To ensure the robustness of the classification, 1000 resampling iterations were conducted, sampling 80% of the patients in each iteration. The optimal number of clusters (k) was rigorously determined by comprehensively evaluating the consensus cumulative distribution function (CDF) curve, the relative change in the area under the CDF curve, and overall clustering stability.

### 2.3. Identification of Differentially Expressed Genes and Functional Enrichment Analysis

To elucidate the underlying molecular mechanism differences between the identified clusters, the “Limma” R package (version 3.52.2) was applied to screen for DEGs at the whole-transcriptome level. Specifically, an empirical Bayes method was utilized to moderate the standard errors of the estimated log-fold changes, thereby enhancing the robustness and statistical power of the test. An adjusted *p*-value < 0.05 and |Fold Change| > 2 were used as significance thresholds [36]. Subsequently, functional annotation of the screened DEGs was performed using Gene Ontology (GO) [37] and Kyoto Encyclopedia of Genes and Genomes pathway enrichment analyses [38] using the “clusterProfiler” R package (version 4.4.4) to investigate their biological roles."

### 2.4. Immune Microenvironment Analysis

The tumor immune microenvironment landscape was reconstructed using the Cell-type Identification By Estimating Relative Subsets of RNA Transcripts (CIBERSORT) R package (version 1.03) to quantify the relative abundance of 22 distinct tumor-infiltrating immune cell subsets in the esophageal cancer samples [39]. Additionally, the ESTIMATE algorithm was employed to compute immune and stromal scores; these scores quantitatively serve as indicators of tumor purity and delineate the distinct cellular composition of the tumor microenvironment.

### 2.5. Drug Sensitivity Analysis

Differences in chemotherapeutic response between the two consensus clusters were assessed by predicting the half-maximal inhibitory concentration (IC50) values for 198 anti-cancer drugs using the “oncopredict” R package (version 0.2) [40]. The prediction was anchored on large-scale pharmacogenomic profiling data obtained from the Genomics of Drug Sensitivity in Cancer 2 (GDSC2) database. Non-parametric Wilcoxon rank-sum tests were performed to statistically identify pharmacological agents with differential efficacy between the subtypes. In this context, a lower IC50 value indicates higher drug sensitivity and greater potential therapeutic efficacy.

### 2.6. Construction and Validation of the Prognostic Model

To identify disulfidptosis-related genes with significant prognostic value, univariate Cox proportional hazards regression analysis was first performed on the previously identified DEGs to screen for robust associations with overall survival. Subsequently, genes exhibiting statistical significance were incorporated into a multivariate Cox regression analysis to isolate independent prognostic factors and eliminate co-linear variables. Based on the regression coefficients (*Coef*) derived from the optimal multivariate model and the expression levels (*Exp*) of the selected genes, a specific risk score formula was constructed to quantify the prognostic risk for each individual patient. The risk score was calculated as follows:

$$Risk\;Score=\sum_{i=1}^{n}\left(Coef_{i}\times Exp_{i}\right)$$

Patients were then systematically classified into high-risk and low-risk groups using the median risk score of the cohort as the predefined cutoff threshold. The predictive accuracy and sensitivity of the signature were evaluated using time-dependent ROC curve analysis via the “timeROC” R package (version 0.4) and pROC (version 1.17.0.1) [41]. Finally, Kaplan–Meier survival analysis coupled with the log-rank test was utilized to compare the long-term survival differences between the high-risk and low-risk groups. These analyses and corresponding survival curves were generated using the “survival” (version 3.3.1) and “survminer” (version 0.4.9) R packages."

### 2.7. Development of a Nomogram

After confirming through multivariate analysis that the five-gene risk score served as an independent prognostic factor, a comprehensive nomogram was constructed using the “rms” R package (version 6.3-0) [42]. This visualization tool strategically integrated the molecular risk score with routine clinicopathological factors, specifically age, gender, and tumor stage, to provide clinicians with precise numerical probabilities for 1-, 3-, and 5-year overall survival. The predictive reliability and discrimination capability of the nomogram were evaluated using ROC curve analysis and Kaplan–Meier survival analysis based on the total nomogram scores.

### 2.8. External Validation

The GSE53625 dataset [43] from the GEO database was used for external validation. The TCGA training cohort includes both adenocarcinoma and esophageal squamous cell carcinoma. Furthermore, TCGA uses RNA-sequencing. In contrast, the GEO cohort only includes pure squamous cell carcinoma and uses Microarray platforms. Due to these biological and technical differences, the model was refitted. A new multivariate Cox regression was performed using only the pure squamous cell carcinoma patients from TCGA. This created a new subtype-specific risk score formula. This method accurately evaluates the true prognostic value of the five genes. It also effectively avoids the biases caused by different tumor subtypes and sequencing platforms.

## 3. Results

### 3.1. Expression Characteristics of Disulfidptosis-Related Genes in Esophageal Cancer

**Figure 1:**
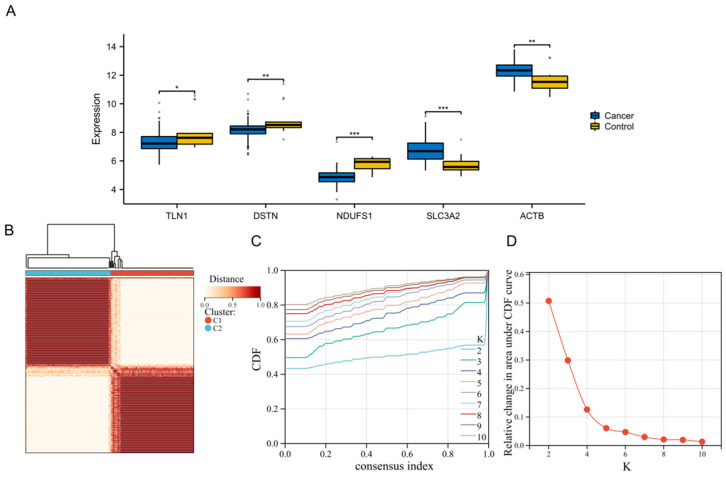
Expression characteristics of disulfidptosis-related genes and consensus clustering analysis in esophageal cancer. (**A**) Box plots detailing the differential mRNA expression levels of five key disulfidptosis-related genes (*TLN1*, *DSTN*, *NDUFS1*, *SLC3A2*, and *ACTB*) between normal control tissues (yellow) and esophageal cancer tissues (blue) from the TCGA cohort. (**B**) Consensus clustering matrix heatmap based on the expression profiles of 23 disulfidptosis-related genes, demonstrating optimal sample segregation when the cluster number is set to k = 2. (**C**) The consensus cumulative distribution function curve, used to assess cluster stability across varying values of k (k = 2 to 10). (**D**) Line graph illustrating the relative change in the area under the CDF curve for varying cluster numbers (k), supporting k = 2 as the optimal cluster number.

A systematic comparison of the expression levels of the 23 disulfidptosis-related genes between tumor tissues and normal control samples revealed significant expression dysregulation in esophageal cancer tissues. Specifically, our analysis revealed global expression dysregulation within the tumor tissues, with five key genes (*TLN1*, *DSTN*, *NDUFS1*, *SLC3A2*, and *ACTB*) showing significant differential expression in esophageal cancer tissues (Figure 1A). Notably, these genes are primarily involved in cytoskeletal structure, mitochondrial metabolism, and transport mechanisms, suggesting their potential roles in the malignant evolution of esophageal cancer.

### 3.2. Identification of Disulfidptosis-Related Molecular Subtypes

Unsupervised consensus clustering was performed using the expression profiles of the 23 evaluated disulfidptosis-related genes. The CDF and clustering stability were utilized to determine the optimal number of clusters. As demonstrated in Figure 1C, the CDF curve exhibited the flattest middle segment and the least dramatic variations when the cluster number was set to k = 2. And the relative change in the area under the CDF curve (Figure 1D) showed the largest drop before leveling off at k = 2, indicating that further increasing the number of clusters would not significantly improve cluster stability. Therefore, the optimal number of clusters was rigorously determined to be k = 2. This stratified the entire esophageal cancer patient cohort into two independent and robust molecular subgroups: Cluster 1 (C1, n = 91) and Cluster 2 (C2, n = 93). Furthermore, as shown in the consensus clustering matrix (Figure 1B), setting the cluster number to k = 2 yielded clear, distinct boundaries with minimal overlap, indicating high intra-cluster consensus and low inter-cluster correlation.

### 3.3. Functional Enrichment Analysis of DEGs Between Subtypes

**Figure 2:**
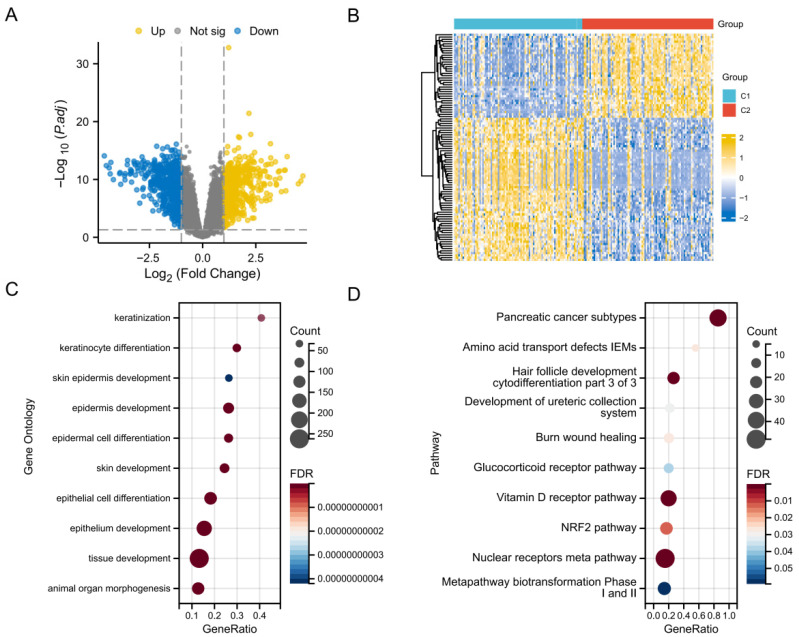
Identification and functional enrichment analysis of differentially expressed genes between two disulfidptosis subtypes. (**A**) Volcano plot visualizing the distribution of DEGs between Cluster 1 and Cluster 2. Yellow dots represent significantly upregulated genes, blue dots represent significantly downregulated genes, and gray dots indicate genes with no significant change (Thresholds: adjusted *p* < 0.05 and |log2 Fold Change| > 1). (**B**) Hierarchical clustering heatmap displaying the distinct expression patterns of the top DEGs between the C1 (blue) and C2 (red) subtypes. (**C**) Bubble plot illustrating the top significantly enriched GO biological process terms for the identified DEGs. The size of the bubble represents the gene count, and the color gradient indicates the False Discovery Rate. (**D**) Bubble plot showing the significantly enriched signaling pathways for the DEGs.

To further investigate the underlying molecular differences between Cluster 1 and Cluster 2, a whole-transcriptome level screening was conducted. A total of 1080 differentially expressed genes (DEGs) were significantly dysregulated between the two subgroups (Supplementary Table S1). The distribution and relative abundance changes in these DEGs were visually depicted via a volcano plot (Figure 2A) and a clustering heatmap (Figure 2B). GO analysis indicated that the DEGs were significantly enriched in cell development-related processes, such as keratinization, keratinocyte differentiation, and skin epidermis development (Figure 2C). Furthermore, pathway enrichment analysis revealed strong associations with pancreatic cancer subtypes, the glucocorticoid receptor pathway, and the NRF2 pathway (Figure 2D). These findings suggest that distinct disulfidptosis patterns may influence the pathophysiological processes of esophageal cancer by regulating specific metabolic and differentiation signaling pathways.

### 3.4. Immune Infiltration Landscape of the Subtypes

**Figure 3:**
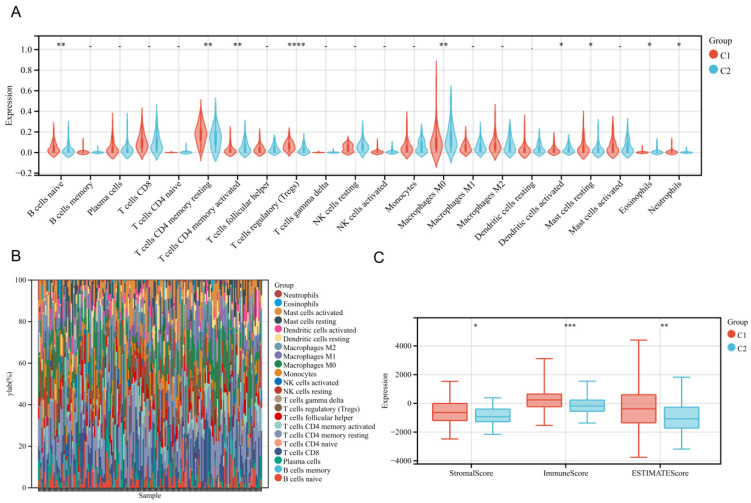
Immune infiltration analysis based on the disulfidptosis-related signature. (**A**) Violin plot comparing the relative abundance of 22 distinct tumor-infiltrating immune cell types between the C1 (red) and C2 (blue) subtypes, quantified using the CIBERSORT algorithm. Statistical differences were assessed using the Wilcoxon rank-sum test. (**B**) Stacked bar chart providing a macroscopic overview of the relative proportions of the 22 immune cell subsets within each individual patient sample across the cohort. (**C**) Box plots comparing the StromalScore, ImmuneScore, and ESTIMATEScore between the C1 and C2 subtypes, calculated using the ESTIMATE algorithm. The C1 subtype exhibits significantly higher scores, indicating a more complex tumor microenvironment.

The tumor immune microenvironment landscape was reconstructed using the CIBERSORT algorithm to quantify 22 distinct infiltrating immune cell subsets. This analysis revealed significant differences in the immune infiltration profiles between the two molecular subtypes (Figure 3A). Compared to Cluster 2, Cluster 1 was characterized by a significantly higher relative abundance of specific immune cells. These included naive B cells, resting and activated CD4 memory T cells, regulatory T cells, and M0 macrophages. To provide a macroscopic overview of this landscape, the relative proportions of these 22 immune cell types within each individual sample were visualized using a stacked bar chart (Figure 3B). This visualization clearly illustrates the overall cellular composition and highlights the profound inter-tumor immune heterogeneity present across the esophageal cancer cohort. Additionally, the ESTIMATE algorithm was employed to evaluate tumor purity and cellular composition. Consistent with the CIBERSORT findings, patients in the C1 subgroup exhibited significantly elevated Stromal, Immune, and ESTIMATE scores relative to the C2 subgroup (Figure 3C). This quantitatively indicates that the C1 subtype possesses a more complex and highly immune-active tumor microenvironment.

### 3.5. Drug Sensitivity Profiles of the Subtypes

**Figure 4:**
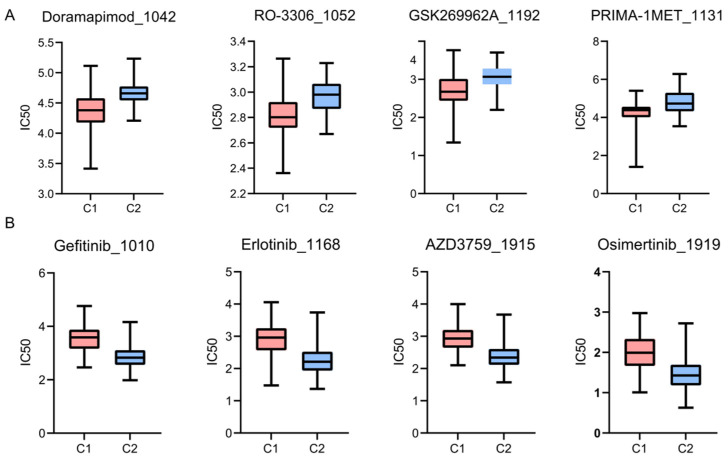
Prediction of differential sensitivity to chemotherapy and targeted drugs between the two subtypes. (**A**) Representative drugs showing lower half-maximal IC50 values in the Cluster 1 subtype. (**B**) Representative drugs showing lower IC50 values in the Cluster 2 subtype.

Differences in chemotherapeutic response between the two clusters were evaluated by predicting the half-maximal inhibitory concentration (IC50) for 198 anti-cancer agents. This drug sensitivity analysis demonstrated distinct therapeutic vulnerabilities specific to each subgroup. Using the Wilcoxon rank-sum test, the C1 cluster exhibited significantly higher sensitivity to several compounds, indicated by lower IC50 values. These included Doramapimod, RO-3306, GSK269962A, and PRIMA-1MET, all *p* < 0.05 (Figure 4A). In contrast, the C2 cluster showed significantly enhanced responsiveness to EGFR-targeted therapies. Highly effective agents for C2 included Gefitinib, Erlotinib, AZD3759, and Osimertinib, all *p* < 0.05 (Figure 4B). These divergent sensitivity profiles highlight the potential utility of disulfidptosis-related subtyping in guiding precision medicine strategies.

### 3.6. Construction of a Five-Gene Prognostic Signature

**Figure 5:**
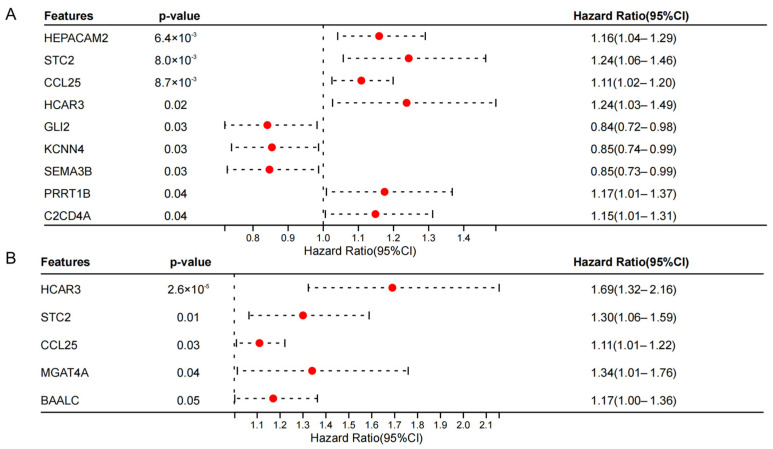
Screening and identification of prognosis-related disulfidptosis genes in esophageal cancer. (**A**) Forest plot detailing the results of the univariate Cox regression analysis, highlighting the candidate genes significantly associated with overall survival. The red dots represent the Hazard Ratio, and the dashed lines represent the 95% confidence interval. (**B**) Forest plot displaying the results of the subsequent multivariate Cox regression analysis, which isolated *HCAR3*, *STC2*, *CCL25*, *MGAT4A*, and *BAALC* as five independent prognostic genes used to construct the final risk model.

To identify specific disulfidptosis-related genes with significant prognostic value, a univariable Cox regression analysis was first performed on the DEGs. This initial screening identified nine candidate genes that were significantly correlated with patient overall survival (Figure 5A). Subsequently, a multivariate Cox regression analysis was applied to isolate independent prognostic factors. This narrowed the candidates down to a robust five-gene signature: *HCAR3*, *STC2*, *CCL25*, *MGAT4A*, and *BAALC* (Figure 5B). Based on the specific regression coefficients derived from this multivariate model, a risk score formula was constructed to quantify individual patient risk. The formula was established as follows: Risk score = (0.507 × *HCAR3*) + (0.198 × *STC2*) + (0.098 × *CCL25*) + (0.243 × *MGAT4A*) + (0.114 × *BAALC*).

### 3.7. Performance of the Prognostic Model

**Figure 6:**
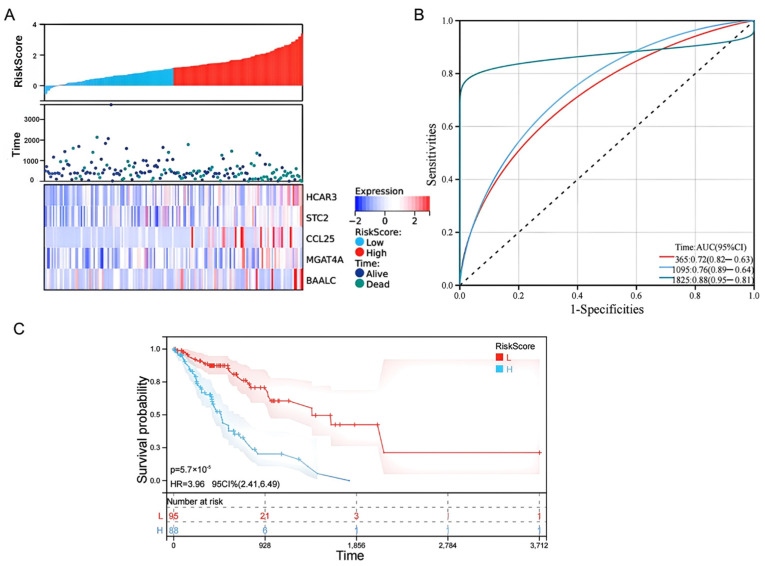
Construction and performance evaluation of the 5-gene prognostic risk scoring model. (**A**) Top panel: distribution curve of the calculated risk scores for patients in the low-risk (blue) and high-risk (red) groups. Middle panel: scatter plot illustrating patient survival status and survival time relative to their risk score. Bottom panel: heatmap demonstrating the expression profiles of the five signature genes across the low- and high-risk groups. (**B**) Time-dependent ROC curves evaluating the model’s predictive accuracy. The AUC values are provided for 1-year (red), 3-year (blue), and 5-year (green) overall survival. (**C**) Kaplan–Meier survival curves demonstrating that patients stratified into the low-risk group (red line) exhibit significantly prolonged overall survival compared to those in the high-risk group (blue line).

Patients within the cohort were classified into high-risk and low-risk groups using the median derived risk score as the predefined cutoff threshold. The distribution of these risk scores, paired with patient survival status and expression heatmaps of the five signature genes, illustrated a distinct and clear separation between the two groups (Figure 6A). The predictive accuracy of this risk model was then evaluated utilizing time-dependent ROC analysis. The model demonstrated excellent discrimination capabilities across multiple timepoints. The calculated area under the curve (AUC) values were 0.72 for 1-year, 0.76 for 3-year, and 0.88 for 5-year survival (Figure 6B). Finally, Kaplan–Meier survival analysis coupled with the log-rank test was utilized to compare clinical outcomes. This revealed that patients stratified into the low-risk group exhibited significantly prolonged overall survival compared to those in the high-risk group (Figure 6C).

### 3.8. Development and Validation of a Nomogram

**Figure 7:**
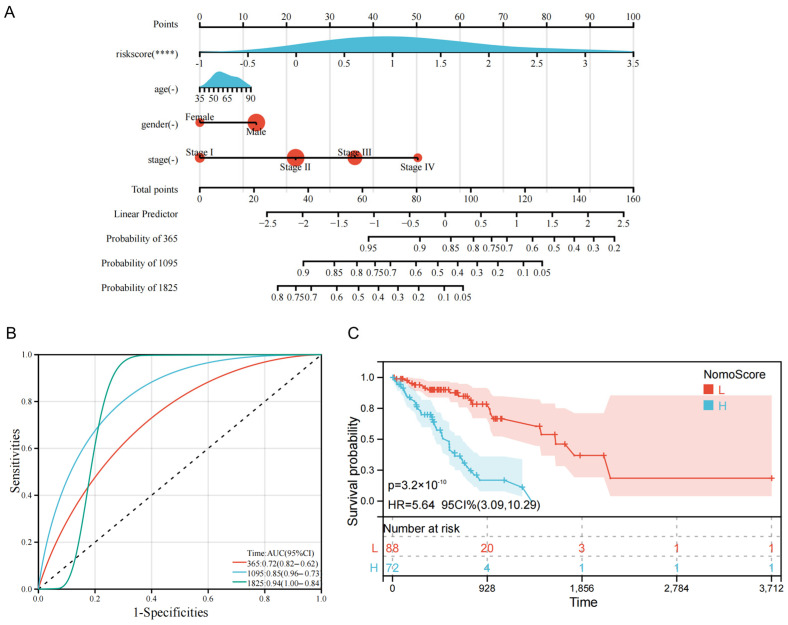
Construction and validation of a nomogram integrating risk score and clinical features. (**A**) The developed nomogram designed to predict the 1-, 3-, and 5-year overall survival probabilities for individual esophageal cancer patients. The tool integrates the 5-gene risk score with patient age, gender, and tumor stage to generate a Total Points score. (**B**) Time-dependent ROC curves assessing the predictive accuracy and discrimination capability of the comprehensive nomogram for 1-, 3-, and 5-year survival. (**C**) Kaplan–Meier survival curves based on patient stratification using the total nomogram score, confirming that lower total scores correlate with significantly better survival outcomes.

Following confirmation that the five-gene risk score functioned as an independent prognostic factor, a comprehensive clinical nomogram was constructed. This visualization tool integrated the calculated risk score alongside established clinicopathological factors, specifically patient age, gender, and tumor stage (Figure 7A). The objective was to provide clinicians with precise numerical probabilities for individual 1-, 3-, and 5-year overall survival. The nomogram’s predictive reliability was systematically evaluated using ROC curve analysis. The tool demonstrated exceptional predictive accuracy, achieving robust AUC values of 0.72, 0.85, and 0.94 for 1-, 3-, and 5-year survival, respectively (Figure 7B). Survival analysis conducted based on total nomogram scores further validated its clinical efficacy. It confirmed that patients accumulating lower total points experienced significantly better long-term survival outcomes (Figure 7C). This robust performance supports the nomogram as a powerful and reliable tool for individualized risk assessment in esophageal cancer.

### 3.9. External Validation in the GSE53625 Cohort

**Figure 8:**
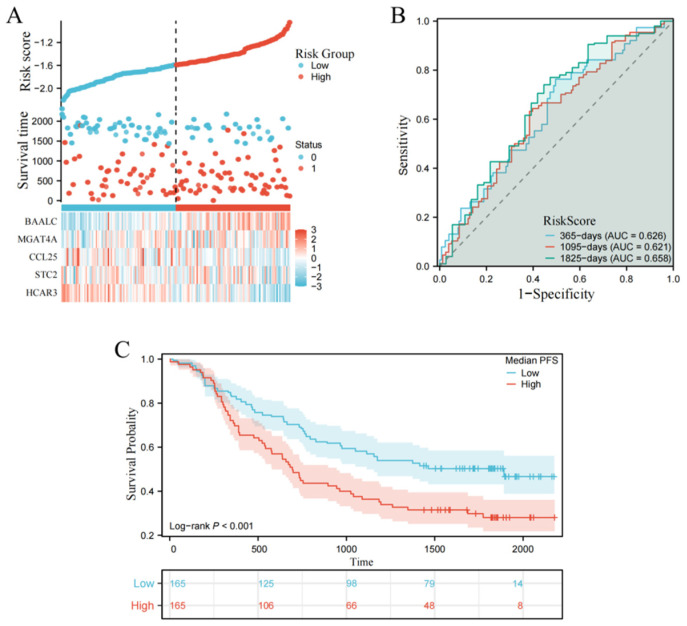
External validation of the 5-gene prognostic risk scoring model in the GSE53625 cohort. (**A**) Top panel: distribution curve of the calculated risk scores for patients stratified into the low-risk and high-risk groups. Middle panel: scatter plot illustrating patient survival status and survival time relative to their risk score. Bottom panel: heatmap demonstrating the expression profiles of the five signature genes (*HCAR3*, *STC2*, *CCL25*, *MGAT4A*, and *BAALC*) across the low- and high-risk groups. (**B**) Time-dependent ROC curves evaluating the model’s predictive accuracy in the external validation cohort. The AUC values are provided for 1-year (AUC = 0.626), 3-year (AUC = 0.621), and 5-year (AUC = 0.658) overall survival. (**C**) Kaplan–Meier survival curves demonstrating that patients stratified into the low-risk group exhibit significantly prolonged overall survival compared to those in the high-risk group.

The five-gene signature was validated using the GSE53625 cohort. To address the platform and subtype differences, the refitted ESCC-specific formula was applied: Risk Score = (−0.138 × *HCAR3*) + (−0.034 × *STC2*) + (−0.073 × *CCL25*) + (0.027 × *MGAT4A*) + (0.102 × *BAALC*). Similar to the TCGA cohort, the GSE53625 patients were divided into high-risk and low-risk groups. Figure 8A shows the risk score distribution, survival status, and gene expression heatmaps. The time-dependent ROC curves showed AUC values of 0.626, 0.621, and 0.658 for 1-, 3-, and 5-year overall survival (Figure 8B). More importantly, the Kaplan–Meier analysis showed a significant difference between the two groups. Patients in the low-risk group lived much longer than those in the high-risk group (Log-rank *p* < 0.001, Figure 8C). These results prove that the five-gene signature is reliable across different datasets.

## 4. Discussion

Esophageal cancer persists as a formidable oncological challenge due to its aggressive trajectory, early lymphatic dissemination, and persistently dismal prognosis. Furthermore, patients with identical TNM stages often experience radically divergent clinical outcomes. This divergence suggests that survival and therapeutic resistance depend not only on tumor size or spread but also on intrinsic metabolic wiring and tumor microenvironment interactions.

The primary objective of this study was to transcend the limitations of anatomical staging by interrogating the transcriptomic landscape of esophageal cancer through the lens of disulfidptosis, a recently delineated form of regulated cell death. By using transcriptome data from TCGA databases, we constructed a 5-gene prognostic risk model that stratified patients into high-risk and low-risk groups with significantly different overall survival outcomes. A nomogram, including clinical characteristics and risk prognostic model scores, was constructed and shown to perform well.

The initial identification of 1080 differentially expressed disulfidptosis-related genes between the two esophageal cancer patient clusters provides a comprehensive molecular portrait of the dysregulated pathways underlying this form of regulated cell death in esophageal tumor occurrence. Functional enrichment analysis of these DEGs revealed significant associations with pathways including epidermal cell differentiation, the NRF2 pathway, pancreatic cancer subtypes, and the glucocorticoid receptor pathway. The enrichment of epidermal differentiation pathways suggests that disrupted epithelial maturation may contribute to esophageal carcinogenesis, potentially linking to epithelial–mesenchymal transition and cancer stemness. Similarly, activation of the NRF2 pathway, a known regulator of redox homeostasis and ferroptosis resistance, is a recognized hallmark of disulfidptosis. Finally, the enrichment of the glucocorticoid receptor pathway suggests a role for dysregulated steroid hormone signaling, as glucocorticoids are known to influence ferroptosis sensitivity, implying a complex interplay between hormone homeostasis and redox regulation.

The CIBERSORT analysis revealed significant differences in the immune cell infiltration patterns between the different clusters as defined by the 5-gene prognostic signature. Specifically, the C1 cluster exhibited a higher abundance of immunosuppressive cell types such as naive B cells, resting memory CD4+ T cells, and regulatory T cells compared to the C2 cluster. These cell types are known to inhibit antitumor immune responses and promote a tumor-permissive microenvironment, which may contribute to the poorer clinical outcomes observed in the C1 cluster patients.

We also observed higher StromalScore, ImmuneScore, and ESTIMATEScore in the C1 cluster. The elevated scores in the C1 cluster potentially represent the activation of various biological processes in the tumor microenvironment, including angiogenesis, extracellular matrix organization, and immune evasion mechanisms. The immunosuppressive phenotype associated with the C1 cluster likely creates a more favorable niche for tumor growth and metastasis, ultimately leading to the observed poorer prognosis. Furthermore, we evaluated the sensitivity of the two clusters to various chemotherapeutic agents. The analysis revealed that the C2 cluster showed significantly enhanced responsiveness to EGFR-targeted therapies, including Gefitinib, Erlotinib, AZD3759, and Osimertinib. Conversely, the C1 cluster exhibited significantly higher sensitivity to compounds such as Doramapimod, RO-3306, GSK269962A, and PRIMA-1MET. The two subtypes show different therapeutic vulnerabilities. This is hypothetically due to their distinct molecular and microenvironmental landscapes. The core mechanism of disulfidptosis involves aberrant disulfide crosslinking of actin cytoskeleton proteins. This ultimately leads to cytoskeleton collapse. The C1 cluster is highly sensitive to certain inhibitors. This might be linked to its inherently more complex tumor microenvironment, marked by higher Stromal and Immune scores. In contrast, the C2 cluster is vulnerable to EGFR inhibitors. This suggests a specific metabolic dependency. However, the exact biological reasons remain speculative at this stage. These drug sensitivities are predictive computational estimates, not validated mechanisms.

Subsequently, the 1080 disulfidptosis-related DEGs were further evaluated using univariable and multivariate Cox regression analyses to establish a prognostic risk model. This integrative approach led to the identification of a 5-gene (*HCAR3*, *STC2*, *CCL25*, *MGAT4A*, *BAALC*) signature that could effectively stratify esophageal cancer patients into high-risk and low-risk groups based on their disulfidptosis-related molecular profile. The high-risk group identified by this model exhibited significantly poorer overall survival compared to the low-risk group, underscoring the clinical relevance of disulfidptosis-related molecular alterations in esophageal cancer progression and prognosis. The establishment of this disulfidptosis-related prognostic signature not only advances our understanding of the molecular underpinnings of esophageal cancer but also provides a potential biomarker panel for risk stratification and personalized therapeutic management of this highly aggressive malignancy. To further contextualize the clinical relevance of our model, it is essential to explore the biological roles of the five signature genes and their specific implications in esophageal cancer progression and disulfidptosis mechanisms. *HCAR3* is a metabolite-sensing G-protein-coupled receptor. It regulates cellular energy metabolism and epithelial proliferation under metabolic stress. Disulfidptosis is fundamentally triggered by glucose starvation. Therefore, *HCAR3* may play a key role in the metabolic reprogramming that determines tumor cell susceptibility to disulfide stress. *STC2* is a stress-responsive glycoprotein consistently upregulated in gastrointestinal malignancies, including esophageal cancer. It promotes glycolysis, epithelial–mesenchymal transition, and metastasis. *STC2* expression is sharply induced under nutrient deprivation and hypoxia to maintain redox homeostasis. This adaptive mechanism may directly counteract the disulfide stress that drives disulfidptosis. *CCL25* is a chemokine that binds to its receptor CCR9 to drive tumor cell proliferation and invasion in esophageal cancer. Its secretion remodels the tumor microenvironment by recruiting specific immune cell populations. This aligns with our observation of distinct immune infiltration patterns across the disulfidptosis-related clusters. *MGAT4A* is a glycosyltransferase responsible for branched N-glycosylation of key membrane proteins, particularly EGFR. This enzymatic activity stabilizes EGFR signaling and promotes tumor invasion. Notably, our C2 cluster showed enhanced responsiveness to EGFR-targeted therapies. *MGAT4A*-mediated regulation of EGFR thus provides a functional link between disulfidptosis profiles and targeted therapy vulnerabilities. *BAALC* functions as a cytoplasmic scaffolding protein integrated into the cytoskeleton network. A core mechanism of disulfidptosis involves aberrant crosslinking of actin proteins, leading to cytoskeletal collapse. *BAALC* overexpression in aggressive cancers promotes cell cycle progression and structural integrity. This suggests it may regulate the cytoskeleton to help tumor cells resist disulfidptosis. Collectively, these five genes do not merely serve as statistical prognosticators; they represent critical biological nodes at the intersection of metabolic adaptability, tumor microenvironment remodeling, and cytoskeletal maintenance. Their collective dysregulation provides a mechanistic explanation for the aggressive biological trajectory observed in high-risk esophageal cancer patients. Further investigations into the mechanistic role of these five genes in disulfidptosis regulation and their interactions with other cancer-relevant pathways may uncover novel therapeutic vulnerabilities and guide the development of targeted interventions. The five-gene model was successfully validated in an independent external cohort (GSE53625). The TCGA and GEO datasets utilize different sequencing platforms and encompass distinct tumor subtypes. These differences were adjusted by refitting the formula strictly for squamous cell carcinoma patients. After this adjustment, the model still demonstrated a strong prognostic value (Log-rank *p* < 0.001). The AUC values in the validation cohort (0.621 to 0.658) were slightly lower than those in the training set. This is an expected result when testing data from different platforms and populations. Overall, the external validation proves that the five-gene signature is a robust and reliable tool.

To better contextualize the novelty and clinical relevance of our proposed disulfidptosis-related signature, it is essential to compare its performance with previously published prognostic models in esophageal cancer. While several existing models have primarily focused on autophagy-related [44], ferroptosis-related [45], or immune-related gene sets [46], our study pioneers the application of a specifically disulfidptosis-driven gene signature. Prognostic models based on traditional pathways typically report moderate predictive accuracies. For instance, the 1-year AUCs for the aforementioned autophagy-related and ferroptosis-associated signatures were reported as 0.746 [44] and 0.656 [45], respectively, while the immune-related signature reported an AUC of 0.785 in its combined cohort [46]. In contrast, our five-gene risk model and its integrated nomogram demonstrated superior long-term predictive accuracy, achieving a 5-year AUC of 0.88 for the risk score alone and an outstanding AUC of 0.94 for the integrated nomogram. This comparison underscores the unique prognostic value and translational potential of targeting disulfide stress mechanisms over conventional cell death pathways.

However, despite the promising clinical implications of our findings, several limitations of the present study should be acknowledged. Firstly, our study relies primarily on retrospective data from TCGA, which represents a specific patient demographic. However, we successfully addressed the potential risk of overfitting associated with the relatively modest sample size of the TCGA-ESCA cohort through both internal and external validation. To assess the initial robustness of our prognostic signature, we implemented rigorous internal validation strategies, including 1000 resampling iterations during consensus clustering and evaluating the stability of coefficients during multivariate Cox regression. The consistent and robust AUC values achieved internally suggested that the model captures genuine biological signals rather than random noise. Crucially, we further validated the five-gene model in an independent external cohort, which proved that the signature is a robust and reliable tool across different datasets and platforms. Nevertheless, we acknowledge that the lack of a large-scale, prospective, multi-center validation cohort limits the immediate generalization of the findings to diverse global populations, particularly given the etiological differences between Eastern and Western esophageal cancer cases. Therefore, future studies should focus on validating this five-gene signature in prospective clinical trials to fully confirm its real-world clinical utility. Secondly, as a bioinformatics-driven analysis, there is an inherent biological gap between mRNA expression levels and actual functional protein activity. The mechanistic links proposed herein are derived entirely from transcriptomic associations and literature synthesis. Similarly, the drug sensitivity profiles and immune infiltration landscapes are based solely on computational algorithms (e.g., CIBERSORT) and pharmacogenomic databases. Furthermore, because computational estimates of immune cell composition can naturally vary depending on the specific analytical methods and reference matrices employed, relying solely on a single deconvolution approach represents an additional limitation. While these computational tools generate highly valuable clinical hypotheses, they lack direct experimental confirmation. Direct biological evidence, such as CRISPR-Cas9 knockout of the signature genes, patient-derived organoid drug screening, single-cell RNA sequencing to resolve precise immune cell topography, and in vivo xenograft models, is currently absent from this report. Therefore, transitioning these findings from the “dry-lab” to the “wet-lab” through rigorous in vitro and in vivo functional assays is an indispensable next step to validate the translational relevance of this five-gene signature. A notable methodological consideration in our study is the stratification of the esophageal cancer cohort into only two disulfidptosis-related subtypes. The TNM staging system relies primarily on anatomical features and fails to reflect the intrinsic molecular heterogeneity of tumors. Given the profound multi-omics heterogeneity of esophageal cancer, a simple two-group classification may oversimplify this complexity and miss the full range of subclonal variations among patients. However, our grouping specifically captures the transcriptomic changes driven by 23 key disulfidptosis genes. This k = 2 classification successfully identified two distinct phenotypes. These groups showed significant differences in gene expression, immune infiltration, and stromal scores. This clear division effectively served our primary goal of building a robust prognostic model. Future studies should explore this complexity further. Single-cell RNA sequencing or spatial transcriptomics would be required to unravel the nuanced, multi-layered heterogeneity of disulfidptosis in esophageal cancer.

This study represents a pioneering effort to map the landscape of disulfidptosis in esophageal cancer. By establishing a five-gene prognostic signature, we have provided a novel tool for risk stratification that goes beyond traditional staging. The identification of *HCAR3*, *STC2*, *CCL25*, *MGAT4A*, and *BAALC* as key prognosticators highlights the importance of metabolic adaptability and cytoskeletal integrity in tumor survival.

## 5. Conclusions

This study successfully developed an effective five-gene prognostic signature related to disulfidptosis regulation in esophageal cancer. This disulfidptosis-related prognostic signature holds great promise to not only improve risk stratification and personalized management of esophageal cancer patients but also advance our understanding of the role of disulfidptosis in the pathogenesis and progression of this highly aggressive malignancy. Ultimately, this work may inform the development of novel targeted therapies that exploit disulfidptosis-related vulnerabilities in esophageal cancer.

## Figures and Tables

**Table 1:** Detailed functional annotation of disulfidptosis-related genes in esophageal cancer.

Functional Cluster	Gene Symbol	Official Gene Name	Primary Biological Function
Transporters	*SLC7A11*	Solute Carrier Family 7 Member 11	Cystine/Glutamate Antiporter [13]
	*SLC3A2*	Solute Carrier Family 3 Member 2	Chaperone for SLC7A11; Integrin signaling [14]
Cytoskeleton Structure	*ACTB*	Actin Beta	Cytoskeletal filament [15]
	*ACTN4*	Actinin Alpha 4	Actin bundling protein [16]
	*FLNA*	Filamin A	Actin cross-linking scaffold [17]
	*FLNB*	Filamin B	Actin cross-linking scaffold [18]
	*TLN1*	Talin 1	Integrin-actin linker [19]
	*IQGAP1*	IQ Motif Containing GTPase Activating Protein 1	Scaffold for Rho GTPases [20]
	*CD2AP*	CD2 Associated Protein	Actin stabilization/Endocytosis [21]
	*PDLIM1*	PDZ and LIM Domain Protein 1	Cytoskeletal adaptor [22]
	*CAPZB*	Capping Actin Protein Beta	Actin filament capping [23]
	*DSTN*	Destrin	Actin depolymerizing factor
Motor Proteins	*MYH9*	Myosin Heavy Chain 9 (Myosin IIA)	Contractile motor protein [24]
	*MYH10*	Myosin Heavy Chain 10 (Myosin IIB)	Contractile motor protein [25]
	*MYL6*	Myosin Light Chain 6	Myosin regulator [26]
Regulators	*NCKAP1*	NCK Associated Protein 1	WAVE complex component [27]
	*INF2*	Inverted Formin 2	Formin (Actin nucleator) [28]
Metabolism	*GYS1*	Glycogen Synthase 1	Glycogen synthesis [29]
	*NDUFS1*	NADH Dehydrogenase Fe-S Protein 1	Mitochondrial Complex I [30]
	*NDUFA11*	NADH Dehydrogenase Subunit A11	Mitochondrial Complex I [31]
	*NUBPL*	Nucleotide Binding Protein Like	Complex I assembly factor [32]
	*LRPPRC*	Leucine Rich PPR Containing	Mitochondrial RNA stability [33]
ER/Stress	*RPN1*	Ribophorin I	N-glycosylation (OST complex) [34]

## Data Availability

All transcriptomic and clinicopathological data are publicly available on http://portal.gdc.cancer.gov/ (accessed on 1 September 2024). GSE53625 is available on https://www.ncbi.nlm.nih.gov/geo/ (accessed on 20 March 2026).

## Supplementary Materials

The following supporting information can be downloaded at https://www.mdpi.com/article/10.3390/biology15070545/s1. Table S1. The details of 1080 DEGs between disulfidptosis-related clusters.
